# The effect of soft tissue augmentation on the clinical and radiographical outcomes following immediate implant placement and provisionalization: a systematic review and meta-analysis

**DOI:** 10.1186/s40729-021-00365-4

**Published:** 2021-08-26

**Authors:** Paolo De Angelis, Paolo Francesco Manicone, Edoardo Rella, Margherita Giorgia Liguori, Silvio De Angelis, Sileno Tancredi, Antonio D’Addona

**Affiliations:** 1 Division of Oral Surgery and Implantology, Department of Head and Neck, Oral Surgery, and Implantology Unit, Institute of Clinical Dentistry, Fondazione Policlinico Universitario A. Gemelli IRCCS - Università Cattolica del Sacro Cuore, Rome, Italy; 2 Department of Head and Neck, Oral Surgery and Implantology Unit, Institute of Clinical Dentistry, Catholic University of the Sacred Heart, Gemelli University Polyclinic Foundation, Rome, Italy; 3 Private Dental Practice, Ascoli Piceno, Italy

**Keywords:** Dental implants, Soft tissue augmentation, Connective tissue

## Abstract

**Background:**

Nowadays, due to the esthetic and social demands of patients, conventional staged protocols seem to be increasingly replaced by faster, one-step protocols. The purpose of the present systematic review is to assess the peri-implant soft tissue changes after immediate implant placement and provisionalization (IIPP) comparing patients treated with or without a sub-epithelial connective tissue graft (SCTG) when replacing a single tooth in the esthetic region.

**Methods:**

The present systematic review was written following the PRISMA checklist. Immediate implants placed with a connective tissue graft and without one were compared. The researched primary outcomes were the mid-buccal mucosa level (MBML) facial soft tissue thickness (FSTT) and marginal bone loss (MBL). The weighted mean differences (WMD) were estimated for all three outcomes.

**Results:**

The change in the mid-buccal mucosa level in the intervention group was significantly higher (WMD 0.54; 95% CI 0.33–0.75), with no indication of heterogeneity (I^2^ = 16%). The facial soft tissue thickness increased significantly in the intervention group (WMD 0.79; 95% CI 0.37–1.22). The marginal bone loss was significantly higher in the control group (WMD 0.13; 95% CI 0.07–0.18), with no indication of heterogeneity (I^2^ = 0%).

**Conclusions:**

The results of the meta-analyses showed a statistically significant reduced change of the marginal bone loss and vestibular recession, as well as higher soft tissue thickness, when a graft was used. The included studies had a short observation time; therefore, studies with longer follow-ups are needed to confirm these findings.

## Background

Nowadays, due to the esthetic and social demands of patients, conventional staged protocols seem to be increasingly replaced by faster, one-step surgical protocols [1–3]. In implant surgery, immediate loading of the implant also eliminates the need for second-stage surgery, thereby reducing the patient’s discomfort [2]. Additionally, the placement of a fixture and temporary restoration on the same day of tooth extraction offers esthetic, psychological, and functional advantages when compared with the use of a provisional removable prosthesis.

In 1978, the protocol of placing implants immediately upon tooth extraction was introduced into clinical practice [4] as an alternative to the standard surgical protocol, for which there is a waiting period of at least 3–6 months [5]. This method has recently been classified as type 1, or immediate implant placement, and is defined as the placement of an implant at the same time as tooth extraction [6], whereas immediate restoration has been defined as any restoration placed within 48 h of implant insertion, without any contact with the opposite dentition in both centric and eccentric occlusions [7].

The concept of immediate implant placement and provisionalization (IIPP) was introduced by Wöhrle in 1998 [8] and has since been proven to be a predictable treatment modality in ideal esthetic situations, with success rates comparable to that of delayed implant placement with delayed prosthetic loading procedures [9–11]. Unfortunately, it is necessary to clarify that that IIPP protocol, despite numerous advantages, such as reduced number of dental appointments, shorter length of treatment, and fewer surgical interventions [12], does not preclude dimensional changes following tooth extractions, both in hard and soft tissues. These unavoidable physiological events can negatively influence the esthetic and functional outcomes of the entire treatment due to the loss of buccal bone [13, 14], mucosal recession [15], or ridge dimensional change [16, 17].

The scientific literature reports that less favorable pink esthetics are not uncommon with immediate implant placement [18–20]. Systematic reviews and clinical studies investigating immediate implant placement and soft tissue volume around implants recommend strict selection criteria, including a thick tissue biotype, which is associated with less mucosal recession compared to a thin mucosa [21], and an intact buccal socket wall to reduce the esthetic risk [18, 22–25]. Additionally, to limit the effects of bone remodeling on the mid-buccal mucosa, it was proposed that implants be placed at least 2 mm from the internal buccal socket wall and to fill the implant–socket gap with a bone graft [26, 27] or that a sub-epithelial connective tissue graft (SCTG) be placed during implant placement to thicken the soft tissue and to obtain satisfactory esthetics [28, 29].

Tissue augmentation procedures with a SCTG have been proven successful for maintaining soft tissue volume and marginal level, and improve the soft tissue thickness [29–31] preserving soft tissue levels when performed in conjunction with implant placement [32–34]. These procedures have also been indicated to facilitate oral hygiene, although the opinion of the scientific literature on this topic remains controversial [35].

However, there is no agreement on the advantages of combining the immediate implant placement with soft tissue grafting, because successful outcomes can be obtained also without soft tissue grafting; furthermore, there is a low level of evidence on the use of xenogenic collagen matrix [16].

However, there is no agreement on the advantages of combining the immediate implant placement with the autogenous soft tissue graft, because successful outcomes can be obtained also without soft tissue grafting; furthermore, there is also a low level of evidence on the use of xenogenic collagen matrix [36].

IIPP does not avoid loss of the buccal bone wall nor mucosal recession or ridge dimensional changes [16, 17].

The purpose of the present systematic review is to assess the peri-implant soft tissue changes (mid-buccal mucosal level, facial soft tissue thickness, and marginal bone loss) after IIPP comparing patients treated with or without a sub-epithelial connective tissue graft when replacing a single tooth in the esthetic region.

## Methods

This systematic review adhered to the Preferred Reporting Item for Systematic Reviews and Meta-Analyses (PRISMA) statement (Moher et al. 2009) [34]. A protocol was written before starting the systematic review and was registered at PROSPERO (University of York, Centre for Reviews and Dissemination) with ID CRD42020181407.

### Clinical question

The clinical question was proposed by following the Participant, Intervention, Comparison, Outcome, Study design (PICOS) principle: “In systemically healthy patients treated by means of single immediate implant placement and provisionalization (participants), with (intervention) or without (comparisons) a simultaneous soft tissue augmentation procedure using a sub-epithelial connective tissue graft, what are the clinical outcomes (primary outcome: mid-buccal mucosal level; secondary outcomes: facial soft tissue thickness and marginal bone loss) reported by retrospective or prospective studies with at least a 12-month follow-up (study design)?

### Study selection

The review was restricted to publications in English or Italian in peer-reviewed journals dealing with patients treated with a single IIPP with or without a soft tissue augmentation procedure in the upper or lower jaw. Only human studies with at least 12 months of follow-up were selected. If articles were reported on case series, at least 10 consecutive cases had to be included. Clinical trials, including randomized controlled trials, prospective and retrospective cohort studies, and prospective and retrospective cases series, were included. The studies must have reported at least 1 of the following outcomes, measured at baseline and after at least 12 months: mid-buccal mucosal level (MBML), facial soft tissue thickness (FSTT), marginal bone loss (MBL).

### Search strategy

The search strategy included the analysis of electronic databases followed by hand searches. A search for relevant studies published in the English language from January 1, 1966, to January 1, 2020, was performed on four databases (MEDLINE, Web of Science, Cochrane Library, Embase) on April 21, 2020, using a search strategy adapted from an original search strategy created for MEDLINE (Appendix 1).

The hand search was conducted to identify relevant studies by screening the reference lists of all full-text articles obtained. We also performed a manual search of journals related to periodontics and implantology, including the *Journal of Dental Research*, *Journal of Clinical Periodontology*, *Journal of Periodontology*, and *International Journal of Periodontics & Restorative Dentistry*.

### Study selection

Following the initial literature search, the titles and abstracts were screened independently by two authors (PDA and ER). Finally, the full text of all studies considered suitable for inclusion by one or both reviewers was obtained to confirm each study’s eligibility based on their fulfilling the inclusion criteria. Disagreements were resolved by a discussion among the reviewers. Data were independently extracted by the two reviewers and recorded in a sheet.

### Quality assessment

The quality of the included studies was assessed using the Cochrane risk of bias assessment tool for randomized studies (RoB 2) following the recommendations included in the Cochrane Handbook for Systematic Reviews of Intervention 5.1.0 (i.e., methods of randomization and allocation concealment, masking of examiners, completeness of the follow-up, selective reporting, and other sources of bias) [11]. Following this assessment, each study was categorized according to the following criteria: (1) low risk of bias (bias unlikely to have seriously altered the data), (2) medium risk of bias (bias could have altered the data; there is some doubt about the correctness of the results), and (3) high risk of bias (there is a serious risk that the findings could have been altered; the strength of the evidence is then seriously weakened). For observational studies, the Newcastle-Ottawa Scale (Appendix 2) adapted by Chambrone et al. and Schepke et al. was used [37, 38]. Each article was evaluated according to 11 methodological quality criteria; 1 star (point) was awarded if an article fulfilled a criterion. Studies with between 11 and 9 stars (approximately ≥ 80% of criteria fulfilled) were considered high-quality; between 8 and 6 stars indicated medium quality, while < 6 stars indicated low quality. Given the small number of studies for each outcome, publication bias was not investigated, following the recommendations of Sterne and Ioannidis [39].

The assessments were performed by two examiners (PFM, MGL). If disagreements occurred in the quality assessment, a third investigator reviewed the study, and a conclusion was reached through discussion.

### Data extraction

The data were independently extracted from the included studies by two authors (SDA, ST). The following parameters were recorded for each study: (1) authors’ names, (2) year of publication, (3) study design, (4) sample size, (5) participants’ demographic information, (6) implant diameter and type, (7) use of bone graft, (8) type of soft tissue augmentation procedure performed, (9) type of provisionalization, (10) protocol of immediate implant positioning, (11) follow-up period, (12) MBML at baseline and 12 months after the surgical procedure, (13) FSTT at baseline and 12 months after the surgical procedure, and (14) MBL at baseline and 12 months after the surgical procedure.

## Statistical analysis

The normality of the variable distribution was assessed using the Shapiro–Wilk test and graphical methods such as Q-Q plot and histogram. The primary outcomes (MBML and FSTT) were calculated as the mean difference between the recorded values at baseline and at the 12-month follow-up. The secondary outcome (MBL) was calculated as the mean difference between the values recorded at the 12-month follow-up and at baseline. In case of the presence of sub-groups in the same arm, the estimates were combined to obtain an overall value according to the Cochrane guidelines [40].

For each endpoint, the estimate of weighted mean difference (WMD) and its relative 95% confidence intervals (CIs) was estimated by pooling the study-specific estimates by random-effect models. These models provided estimates adjusted for potential correlation within studies as well as heterogeneity between studies.

Homogeneity across studies was verified with a test based on Cochran’s Q statistic, which is distributed as a chi-square with k-1 degrees of freedom, where k is the number of studies. Higgins and Thompson’s I^2^ statistic, which ranges from 0 to 100%, was determined to quantify the percentage of total variation across studies that was attributable to heterogeneity rather than to chance. A threshold of I^2^ < 50% was considered an acceptable level of between-study heterogeneity. A forest plot was produced for each outcome to represent the difference graphically.

All the statistical analyses were performed using R version 4.0.2.

## Results

**Fig. 1:**
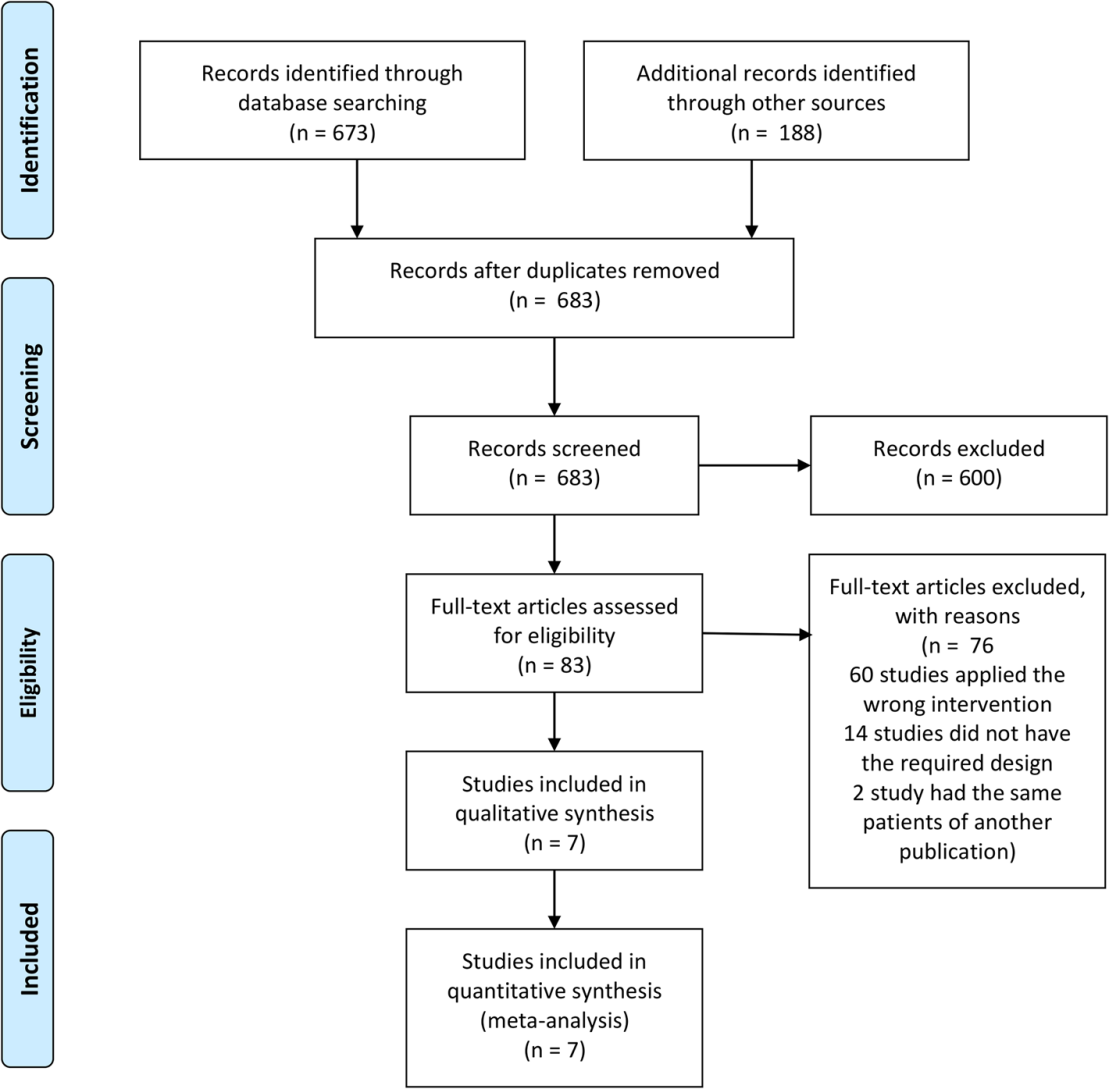
PRISMA flow diagram of the literature search and study selection

The electronic search identified a total of 861 articles. After removing duplicates, the titles and abstracts of 683 articles were analyzed, and based on this analysis, 83 articles were selected for full-text evaluation. After removing 76 articles that did not meet the criteria, a total of seven studies published between 2007 and 2021 were included in this systematic review; the studies involved a total of 272 patients (Fig. 1).

The main features of the studies included in the meta-analysis are summarized in Table 1.

**Table 1:** Characteristics of the included studies

Authors	Acceptor site	Donor site	Number of patients/implants	Study type
Runcharrasaeng	Flapless Subperiosteal	Palate	55	Prospective observational
Yoshino	Flapless Subperiosteal	Palate	20	RCT
Migliorati	Flapless Supraperiostealz	Palate	47	RCT
Noelken	Flapless Supraperiosteal	Palate	26	Retrospective observational
Zuiderveld	Flapless Supraperiosteal	Tuberosity	60	RCT
Frizzera	Flapless Subperiosteal	Palate	16	RCT
De Angelis	Flapless Supraperiosteal	Palate	48	Retrospective observational

**Fig. 2:**
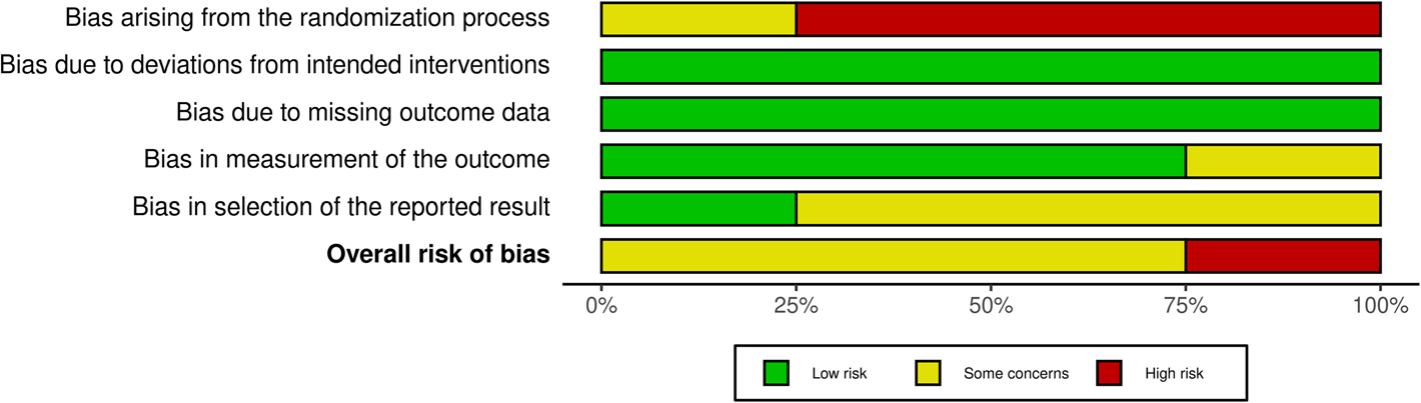
Bar plots of the distribution of the risk of bias judgments within each bias domain

**Fig. 3:**
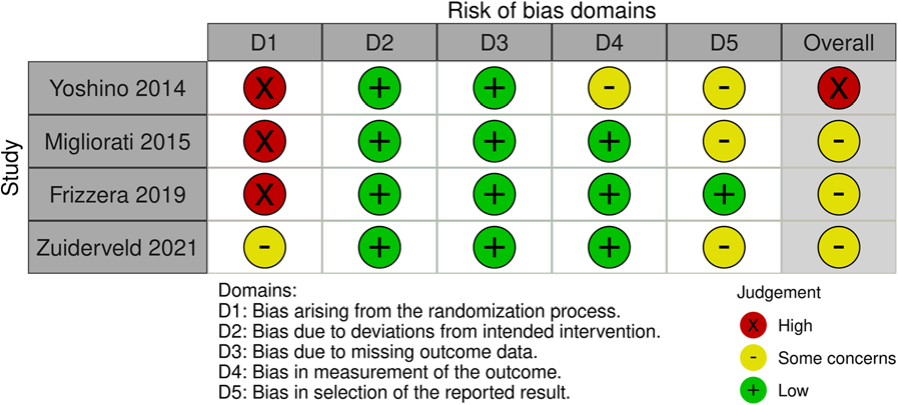
Traffic light plots for each individual result

Four of the selected studies were randomized controlled trials [41–44]; one was a prospective observational study [45], while the remaining two were retrospective observational studies [46, 47] (Figs. 2 and 3). Of these studies, only two [42, 44] recorded all primary and secondary outcomes. Five studies recorded the MBML [41–44, 46] while only four reported the FSTT [42, 44, 45, 47], and four presented the MBL data [41–44].

All surgeries were performed under local anesthesia using a flapless approach, a non-traumatic extraction technique, and debridement of the alveolus. All studies included in the meta-analyses used bone-level implants placed leaving a buccal gap from the bone wall. The implant diameters were 3.3–5 mm.

Autogenous bone [46], a combination of autogenous bone mixed with bone xenograft [43], bone xenograft alone [41, 42, 47] bone xenograft with collagen membrane [44], or a mixture of allograft and xenograft [45] were used to fill the buccal gap. The implants were placed 4 mm apical to the facial gingival margin in one study [44], 3 mm apical in three studies [41, 43, 47],, and were placed 2 mm apical in another study [45].

Two studies did not mention how the implants were positioned in the post-extraction sockets.^42,46.^

For the soft tissue augmentation, five of the studies used an SCTG previously obtained from the palate of the patient [41, 42, 45, 46] or from the maxillary tuberosity [43], while two studies [44, 47] divided the experimental group into two sub-groups: one was treated with a SCTG, while the other was treated with a connective matrix. The SCTG was placed on the buccal side supraperiosteal in three studies [42, 43, 46, 47] and subperiosteal in the other three studies [41, 44, 45].

The provisional restorations were designed to be screw-retained in three studies [43, 44, 47] while one study used a cement-retained design [41] and two utilized both [44, 46] according to the implant position. One study provided no information on the design of the provisional restoration [45]. The provisional restoration was maintained for 6 months in two studies [41, 44] and for 3 months in four studies [42, 43, 46, 47].

After the temporary phase, three studies used a customized zirconia abutment for a cement-retained crown [41, 44, 46], while two studies used a screw-retained or a cement-retained crown on a customized zirconia abutment, depending on the location of the access hole [43, 47] and two studies provided no information on the design of the definitive restoration [42, 45].

A variety of methods were used to measure the soft tissue outcomes, including standardized photographs [43, 44], direct clinical measurements on the patient [45, 46] standardized measurements of the dental casts [41], and standardized photographs of the dental casts [42]. Only one study [43] reported a case of implant loss in both the test and control groups, while all other studies included in the meta-analysis reported a survival rate of 100%.

Table 2 summarizes the outcomes of each study and the details on site and implant characteristics and measuring techniques.

**Table 2:** Clinical and radiographical outcomes of the included studies

Authors	MBML control	MBML test	FSTT control	FSTT test	MBL control	MBL test
Runcharraseng	/	/	0.32 ± 0.36	1.43 ± 0.59		
Yoshino	0.70 ± 0.48	0.25 ± 0.35	/	/	− 0.14 ± 0.53	− 0.01 ± 0.27
Migliorati	0.37 ± 0.68	0.05 ± 0.46	− 0.10 ± 0.71	0.70 ± 1.0	− 0.136 ± 0.107	0.001 ± 0.092
Noelken	1.00 ± 0.92	2.00 ± 0.72	/	/	/	/
Zuiderveld	0.50 ± 1.10	0.10 ± 0.80	/	/	/	/
Frizzera	0.72 ± 0.57	0.04 ± 0.30	1.11 ± 0.63	2.06 ± 0.68	− 0.35 ± 3.69	− 0.55 ± 3.51
De Angelis	/	/	0.21 ± 0.30	0.58 ± 0.45	/	/

### Risk of bias

Regarding the randomization protocol, all trials reported some level of bias: Frizzera et al., Yoshino et al., and Migliorati et al. did not properly report the procedures that allowed them to randomize the enlisted patients properly but merely stated that the sample had been randomized [41, 42, 44]. Therefore, they were defined to be at high risk of bias.

Zuiderveld et al. reported that they adopted the sealed, opaque envelope method of randomizing patients, but no information was added on how the randomization lists were managed, and if those lists were hidden from the operators in charge of enrollment [43]. Therefore, we believe it to be at medium risk of bias.

In all but one study [41], the clinical assessors were blinded to the allocation of patients.

With respect to the selection of the reported result, only Frizzera et al. followed a pre-described plan of analysis [44] while for the other studies, the same plan was not available and could not be obtained: given that, they were deemed to be at medium risk of bias.

**Fig. 4:**
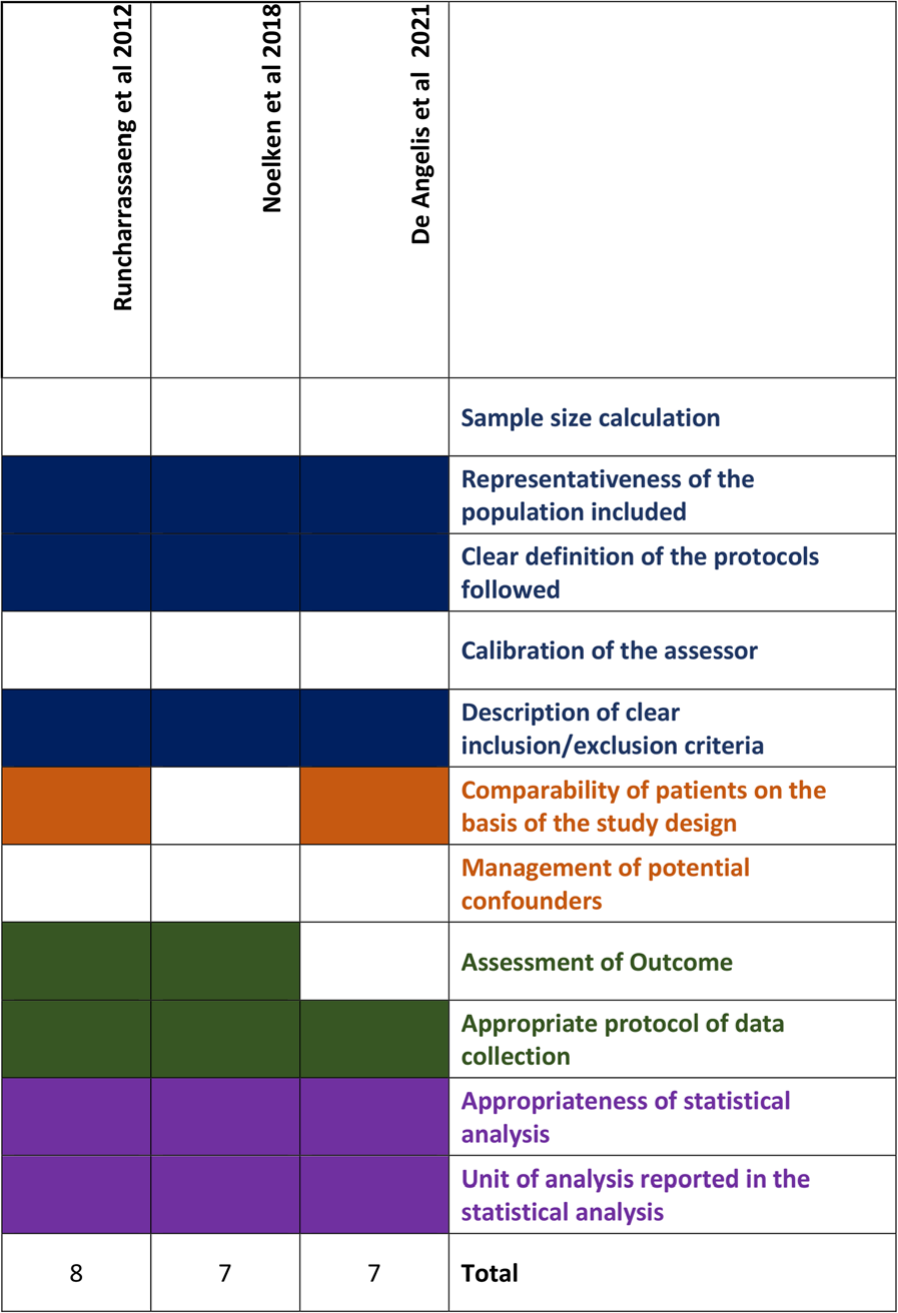
Evaluation of observational studies using the adapted Newcastle-Ottawa Scale

The non-randomized studies were evaluated with the Newcastle-Ottawa Scale; the three studies [45–47] were deemed medium-quality, having received either 7 or 8 stars (Fig. 4); the main risk of bias stemmed from the absence of proper blinding of assessors, and both studies also did not account for the confounding factors that, given the design of both studies, could have been present.

### Synthesis of results

All outcome variables were not normally distributed. The results of WMD estimates are presented in Table 3. There was low heterogeneity in all three research outcomes.

**Table 3:** Meta-analyses results for different outcomes

Endpoint	Estimate	95% CI	p-value	Tau^2^	Q statistic	p-value Q	I^2^
MBML	0.54	0.33–0.75	< 0.001	0.01	4.73	0.32	16.79%
FSTT	0.79	0.37–1.22	< 0.001	0.15	16.33	0.53	82%
MBL	0.13	0.07–0.18	< 0.001	0.00	1.18	0.76	0.00%

**Fig. 5:**
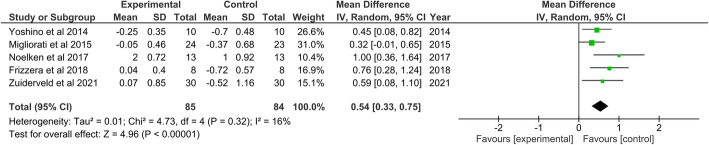
Forest plot for random-effects meta-analysis of mid-buccal mucosa level (MBML). Central squares of each horizontal line represent the weighted mean difference (WMD) for each study. Horizontal lines indicate the range of the 95% CIs; vertical line indicates WMD = 0 (which indicates no difference)

For the MBML, 5 studies were included in the analysis; the MBML in the intervention group was significantly higher on average than that in the control group (WMD 0.54; 95% CI 0.33–0.75; p-value < 0.001), with no indication of heterogeneity (I^2^ = 16%), indicating a higher amount of mucosal recession in the control group than in the intervention group (Fig. 5).

**Fig. 6:**
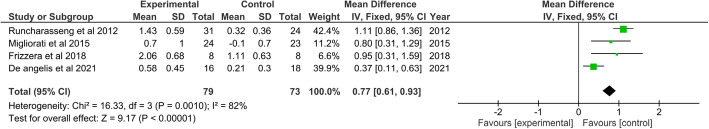
Forest plot for random-effects meta-analysis of facial soft tissue thickness (FSTT). Central squares of each horizontal line represent the weighted mean difference (WMD) for each study. Horizontal lines indicate the range of the 95% CIs; vertical line indicates WMD = 0 (which indicates no difference)

For the FSTT, 4 studies were included in the analysis; the FSTT increased notably in the intervention group, and this increase was significantly higher on average than that in the control group (WMD 0.79; 95% CI 0.37–1.22; p-value < 0.001), indicating that a thicker and more esthetically pleasant soft tissue can be obtained by inserting a SCTG at the time of implant positioning (Fig. 6).

**Fig. 7:**
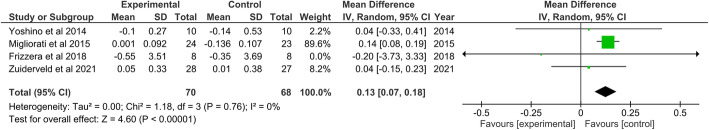
Forest plot for random-effects meta-analysis of marginal bone loss (MBL). Central squares of each horizontal line represent the weighted mean difference (WMD) for each study. Horizontal lines indicate the range of the 95% CIs; vertical line indicates WMD = 0 (which indicates no difference)

For the MBL, 4 studies were included in the analysis; the MBL was significantly higher in the control group than in the intervention group (WMD 0.13; 95% CI 0.07–0.18; p-value < 0.001), with no indication of heterogeneity (I^2^ = 0%), indicating a higher risk for bone resorption in the control group than in the intervention group (Fig. 7).

## Discussion

In the present review, we analyzed and compared the use of single IIPP with or without simultaneous soft tissue augmentation. Soft tissue augmentation procedures are aimed at reducing the risk of peri-implant recessions after immediate implant placement in the esthetic zone [29].

The results of the meta-analyses showed a statistically significant difference for all parameters investigated, outlining better results for the peri-implant marginal recession and marginal bone loss, as well as higher soft tissue thickness, when sub-epithelial connective tissue graft was used.

Among the clinical parameters to be considered before assessing the success of the implant placement are the soft tissue outcomes such as the mid-buccal marginal level of the peri-implant mucosa, the facial soft tissue thickness, the papilla height, and embrasure fill [22]. Due to the strict inclusion criteria of the present review, only a limited number of studies were selected for each clinical parameter. The studies included had homogenous patient inclusion criteria, which were similar. Nevertheless, several clinical parameters were not reported or assessed in the included studies, such as soft tissue phenotype, volume of the papilla, and keratinized tissue width. The results of the present meta-analysis indicate that the use of an SCTG guarantees a statistically significant increase in the stability of the mid-buccal marginal level, with a WMD of 0.55 mm at the 12-month follow-up. These results are in agreement with the systematic review by Lee et al. [29]

In the literature, the absence of a vestibular bone plate and the presence of a thin soft tissue phenotype are considered risk factors for the recession of peri-implant tissues [48]. Among the included studies here, only three reported the preoperative soft tissue phenotype [41–43]. The use of an SCTG can be required in particular in patients with a thin periodontal phenotype, which is usually associated with a thin buccal bone and which in the postoperative phase undergoes greater bone resorption and soft tissue contraction [48]. Yoshino et al. reported that facial level changes observed in the control group (treated without the SCTG) (0.7 mm) were more pronounced than those seen in the test group (treated with the SCTG) (0.25 mm) [41].

Further, Migliorati et al. showed similar values in their RCT, with a mean recession from the initial highness of 0.2 mm when a CTG was performed versus 0.71 mm when no graft was used [42].

In the present study, the MBL value in the grafted group was significantly higher on average than that in the control group. This finding is in line with a recent trial that reported statistically significant less MBL when thick tissues or augmented thin tissues were compared with non-augmented thin tissues [49].

The FSTT was measured in three studies, and a statistically significant gain was observed in the groups treated with SCTG, with a mean difference of 1.04 mm [42, 44, 45].. This is a crucial parameter for peri-implant marginal stability and also from an esthetic point of view. In the anterior area, the primary goal is to recreate a natural restoration, and this requires not only marginal stability but also adequate thickness to maintain the preoperative soft tissue volume. Furthermore, the characteristics of the soft tissue appear to be able to affect peri-implant status health [50]. Although there is no consensus in the scientific literature, some authors support the concept that the lack of keratinized mucosa could jeopardize the maintenance of soft tissue health around dental implants [51].

Greater soft tissue thickness, in addition, can allow the clinician to cover up the grayish shade of the titanium and to obtain patient satisfaction [52].

However, it should be kept in mind that the use of an SCTG could be also associated with the risk of surgical and post-operative complications such as bleeding, swelling, graft necrosis, and patient morbidity as reported by Lee et al. [17] The use of a xenogeneic collagen matrix could be a viable solution for immediate implant placement to avoid these complications, even if the efficacy of this approach still lacks scientific evidence [53].

The studies included in the present review all reported a survival rate of 100%, with only Zuiderveld et al. reporting a failure each in the SCTG-treated group and the no-augmentation group [41–46]. These data outline that there is no difference in terms of survival rate and that osseointegration can be easily achieved and with very high predictability in this challenging clinical condition. However, the success of implant rehabilitation is based on the different parameters in the esthetic area.

One of the limitations of this systematic review is the short-term results considered, which outlines the need for long-term studies to show the differences after the 12-month follow-up between the immediate implants placed with or without soft tissue augmentation. Another limitation is the heterogeneity in the methods used to measure the soft tissue outcomes. Long-term randomized controlled studies are required to confirm the benefits of using SCTG and to clarify the clinical conditions in which soft tissue augmentation for avoiding esthetic complications is advised.

## Conclusions

The results of the meta-analyses showed a statistically significant difference for all parameters analyzed, outlining less peri-implant marginal recession and marginal bone loss, as well as higher soft tissue thickness, when sub-epithelial connective tissue graft was used. Future long-term interventional studies are needed to confirm the above results.

## Data Availability

The datasets used and/or analyzed during the current study are available from the corresponding author on reasonable request.

## Appendix 1

Search strategy

**Table:**

1. (Implant) **OR** (Implantology) **OR** (Dental implantation) **OR** (Implant supported) **OR** (Implant-supported) **OR** (Preprosthetic)	
2. (Graft) **OR** (Augmentation) **OR** (Connective) **OR** (Matrix) **OR** (Biomaterials)	
3. (Post extractive) **OR** (Immediate) **OR** (Immediately) **OR** (Socket)	
4. (Provisional) **OR** (Immediate provisionalization) **OR** (immediate loading)	
5. (Mucosa) **OR** (Mucosal level) **OR** (Gingival level) **OR** (Thickness) **OR** (Papilla) **OR** (Width) **OR** (Bone level)	
6. (#1) **AND** (#2) **AND** (#3) **AND** (#4) **AND** (#5)	
